# Maternal fenvalerate exposure during pregnancy impairs growth and neurobehavioral development in mouse offspring

**DOI:** 10.1371/journal.pone.0205403

**Published:** 2018-10-15

**Authors:** Ji-Jie Liu, Ce Guo, Bo Wang, Meng-Xing Shi, Yang Yang, Zhen Yu, Xiu-Hong Meng, De-Xiang Xu

**Affiliations:** 1 Department of Maternal, Child & Adolescent Health, School of Public Health, Anhui Medical University, Hefei, China; 2 Department of Toxicology, School of Public Health, Anhui Medical University, Hefei, China; Radboud University Medical Centre, NETHERLANDS

## Abstract

Although use of fenvalerate has increased dramatically over the past decade, little is known about their potential adverse effects on growth and development. The purpose of this study was to examine the effects of maternal fenvalerate exposure during pregnancy on growth and neurobehavioral development in the offspring. Pregnant mice were orally administered to fenvalerate (0.2, 2.0, and 20 mg/kg) daily throughout pregnancy. The tests of growth and neurobehavioral development were performed during lactation period. A series of neurobehavioral tasks were carried out from lactation to puberty. Anxiety-related behaviors were evaluated by open-field and elevated plus maze. Morris Water Maze was used to assess spatial learning and memory ability. Results showed that maternal fenvalerate exposure during pregnancy markedly delayed growth development of neonatal offspring during lactation. In addition, anxiety-like behaviors were increased in fenvalerate-exposed male offspring. Moreover, spatial learning and memory was severely impaired in female offspring. Taken together, maternal fenvalerate exposure during pregnancy delayed growth and neurobehavioral development in a gender-dependent manner. Additional study is required to explore the underlying mechanism through which maternal fenvalerate exposure during pregnancy induces impairment of growth and neurobehavioral development.

## Introduction

Fenvalerate, a widely used pyrethroid insecticide, has become a new major public health problem [1]. Several epidemiological reports showed that fenvalerate and its metabolites were detected in bovine milk [2] and human samples, such as breast milk [3] and urine [4]. An investigation found that maternal urinary metabolite levels were about 4–10 times higher than those of general population [5]. More evidences suggest that it should be focus on the impairment induced by fenvalerate exposure. Previous studies have focused on neurotoxicity, reproductive toxicity of fenvalerate [6–8]. In addition, increasing evidence demonstrates that fenvalerate has endocrine disruptive effects [9]. An early study showed that fenvalerate presented weak estrogen activities, strong anti-androgen effect and antagonistic effect on thyroid receptor (TR) signal [10]. According to our laboratory's earlier report, fenvalerate exposure during puberty interfered with the synthesis of testosterone and estradiol during the developing brain [11].

With the emerging paradigm named as “developmental origins of health and disease”, it is becoming important to assess adverse health consequent induced by maternal exposure to fenvalerate during pregnancy. Recently, our research group found that maternal fenvalerate exposure induced fetal intrauterine growth restriction and disrupted placental thyroid hormone receptor signaling [12]. As is well known, the placenta is necessary to maintain the fetal growth and development. Meanwhile, thyroid hormone plays an important role in development of brain and behaviors in early stage of life. However, whether maternal fenvalerate exposure during pregnancy disturbs growth and neurobehavioral development remains unknown.

The purpose of this study was to investigate whether maternal fenvalerate exposure during pregnancy impaired growth and neurobehavioral development in mouse offspring. We found that maternal exposure to fenvalerate during pregnancy delayed growth development. In addition, maternal exposure to fenvalerate during pregnancy impaired spatial cognition and behavioral development in a gender-dependent manner. That is, anxiety-related behaviors were increased in fenvalerate-exposed male offspring. Interestingly, spatial learning and memory were impaired in fenvalerate-exposed female offspring.

## Materials and methods

### Chemicals

Fenvalerate and corn oil were purchased from Sigma Chemical Co. (St. Louis, MO).

## Animals and treatment

The ICR mice (8–10 weeks old; 32–34 g in male mice; 28–30 g in female mice) were purchased from Beijing Vital River whose primary colonies were all introduced from Charles River Laboratories, Inc. The mice were freely allowed to access to food (Beijing Keao Xieli Feed Co, LTD, Beijing 100107) and water at any time. One week prior to use, they were held in a room that was commanded lighting (12 h light/12 h dark cycle), temperature (20–25C) and humidity (50±5%). For mating purposes, four females and two males spend night together starting at 9:00 PM, and females were examined by 7:00 AM the next morning. If a vaginal plug appeared, gestational day (GD) 0 was established. This study was approved by the Association of Laboratory Animal Sciences and the Center for Laboratory Animal Sciences at Anhui Medical University (Permit Number: 13–0012). All procedures on animals followed the guidelines for humane treatment set by the Association of Laboratory Animal Sciences and the Center for Laboratory Animal Sciences at Anhui Medical University (Hefei, China).

To investigate the effects of fenvalerate on neurobehavioral development in offspring mice, twenty-four pregnant mice were randomly divided into four groups. The Food and Agriculture Organization of the United Nations and the World Health Organization (FAO/WHO, 2009) have together established admissible daily intakes (ADI) of 0.02 mg/kg/day for fenvalerate. Therefore, in the present study, we chosed the dose of 0.2 mg/kg, which is tenfold ADI of fenvalerate, as the lowest dose. On the other hand, our preliminary data showed that no signs of maternal toxicity were observed in dams that were administered with fenvalerate (30mg/kg) during pregnancy [13]. Therefore, the dose of 20 mg/kg, about 1/10 LD50 of the fenvalerate, was chosen as the highest dose in this present study. The pregnant mice were administered with fenvalerate (0.2, 2.0, and 20 mg/kg, dissolved in corn oil) by gavage daily GD0 to GD18. The control mice were orally administered to corn oil daily from GD0 to GD18.

Within 24h after birth, three males and three females pups were kept per dam. The pups were weighted daily from PND2 to PND21. According to previous study [14–15], the tests of growth and neurobehavioral development were performed during lactation period. A battery of behavior tests were performed in mice offspring during early stage of life. In order to assess anxiety-related activities, Open field was carried on PND35, and Elevated plus maze was performed on PND40. To evaluate learning and memory function, Morris water maze was performed from PND45 to PND50.

### Elevated plus maze

Based on the design [16], the maze consisted of four arms (two open without walls and two enclosed by 15.25 cm high walls) 30 cm long and 5 cm wide. Took mouse out of its cage and placed at the junction of the open and closed arms, facing the open arm opposite to where the experimenter was. The timer was set for 5 minutes when the rodent was placed in the maze. The experimenter recorded time on open arms and times on open arms. An open arm entry was counted when all four paws of the rodent were on the open arm. The observer must avoid unnecessary movements and making noise. Cleaned the elevated plus maze before testing with another rodent. An increase in open arm activity (duration and/or entries) reflected anti-anxiety behavior.

### Open field

To assess spontaneous movement and anxiety, the open field test was designed [17]. The open-field device was an enclosed arena box with 72 cm long, 72 cm wide and 28 cm high. The digital camera was fixed above the box. The black box floor was divided into 16 equal squares by software. The mice were placed in a center square for 3 min. The indicators recorded includes total number of squares crossed, the number of rearing, the number of grooming, the frequency of feces, the moving speed and the moving distance. After each test, the enclosure was cleaned with water.

### Morris water maze

Morris water maze was designed as a method to assess spatial learning and memory [18]. The apparatus comprised a circular black tank (150 cm in diameter and 30cm high). The tank was abounded with water and the water level was 30 cm in height. A black platform (10 cm in diameter) was used as escape target diving into the water. Water temperature was slightly warmer than ambient air temperatures in the laboratory (19–22°C). Room lighting was indirect. The test consisted of two parts: place navigation and spatial probe. During five consecutive days, place navigation was conducted with four trials per day. On the sixth day, spatial probe was given to assess spatial memory. A video camera, computer and tracking software were used to record performance, including latency swimming distance, swimming speed and frequencies of crossing objective quadrant.

### Data analysis

All data was presented as mean±SEM. Data of place navigation in Morris Water Maze was analyzed by two-way ANOVA for repeated measures. Other data was analyzed by two-way ANOVA. The significance level was set at *P*<0.05. All statistical analyses were performed using the statistical package for social sciences (*SPSS*, version 20.0).

## Results

### Food intake and body weight of pregnant mice exposed to fenvalerate during pregnancy

**Fig 1:**
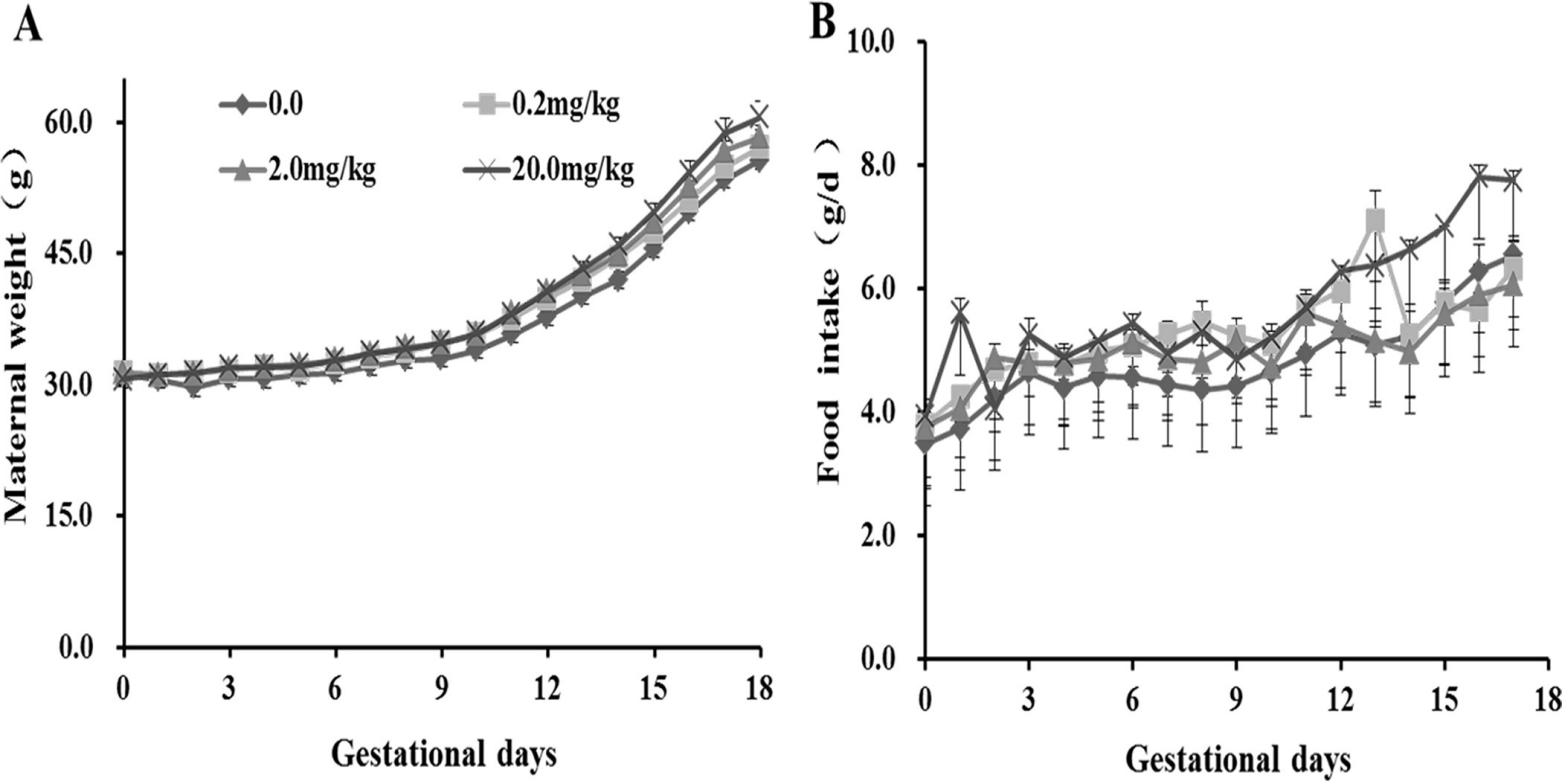
Food intake and body weight of pregnant mice exposed fenvalerate during pregnancy. Pregnant mice were administered with fenvalerate (0, 0.2, 2.0 or 20.0 mg/kg) daily from GD0 to GD18. (A) Food intake of pregnant mice. (B) Maternal weight. All data were expressed as mean± SEM.

Although food intake and body weight of pregnant mice were on an upward trend, there was no significant difference in food intake and body weight among the four groups (Fig 1A and 1B).

### Effects of maternal fenvalerate exposure during pregnancy on growth indexes and neurobehavioral development of neonatal mice during lactation period

**Fig 2:**
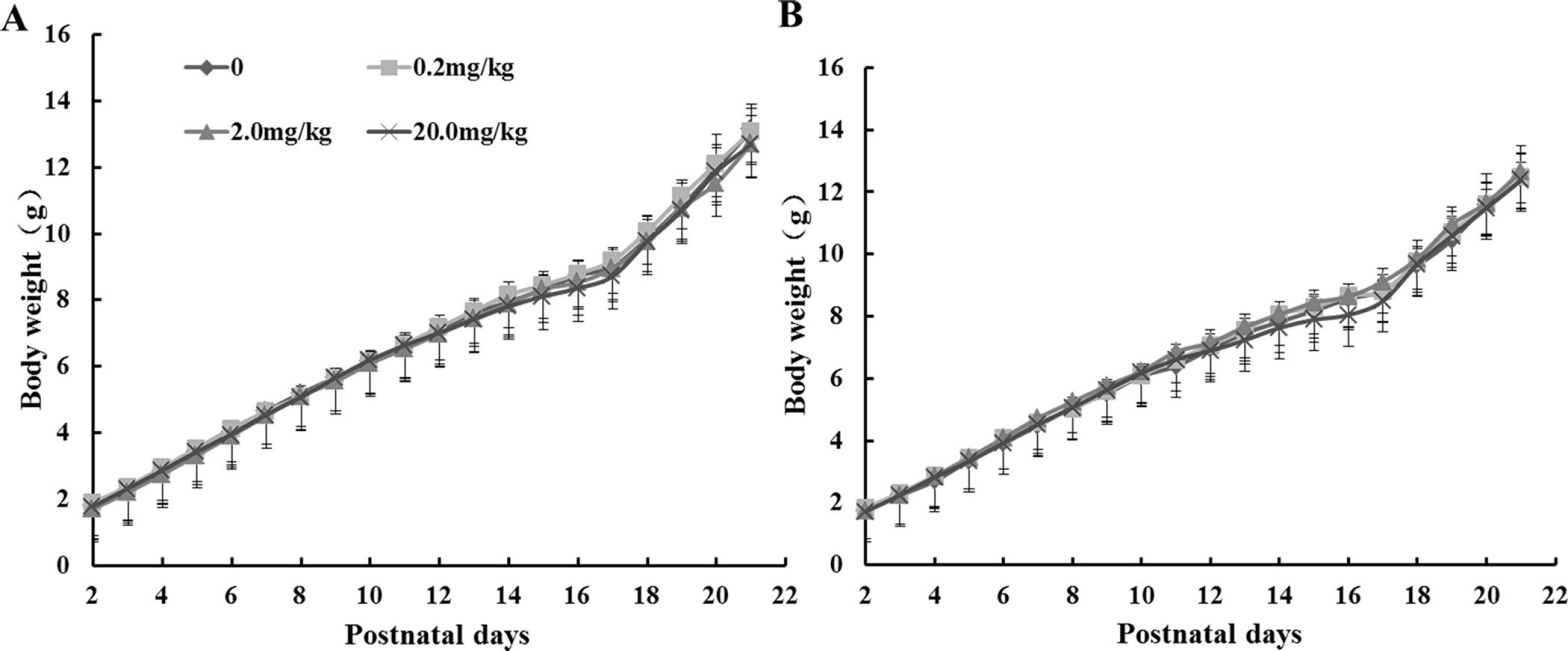
Effects of maternal fenvalerate exposure on body weight of offspring during location. Pregnant mice were administered with fenvalerate (0, 0.2, 2.0 or 20.0 mg/kg) daily from GD0 to GD18. (A) Body weight of male offspring during location. (B) Body weight of female offspring. All data were expressed as mean± SEM.

The effects of maternal fenvalerate exposure during pregnancy on growth indexes of neonatal mice were investigated. First, the effects of maternal fenvalerate exposure during pregnancy on body weight of male and female offspring were analyzed during location. There was no significant difference on body weight was observed among different groups (Fig 2A and 2B). In addition, the effects of maternal fenvalerate exposure during pregnancy on growth maturation and neurobehavioral development of male and female offspring were analyzed during location (Table 1). As shown in Table 1, there was a significant difference in the time of eruption of upper incisors among different groups. Interestingly, the time of eruption of upper incisors was markedly prolonged in offspring with daily exposure to fenvalerate at dose of 20.0 mg/kg. Further analysis revealed that maternal fenvalerate exposure during pregnancy markedly prolonged the time of eruption of upper incisors not only in male groups, but also in female groups (*P<0*.*05*). Neither the time of eruption of lower incisors nor the time of eyes opening was significantly altered among different groups.

**Table 1:** Effects of maternal fenvalerate exposure during pregnancy on growth and neurobehavioral development of neonatal mice during lactation period.

	0	0.2mg/kg	2.0mg/kg	20.0mg/kg	*F*	*P*
**Physiology maturation**						
Eruption of upper incisors	11.56 ± 0.12	11.61 ± 0.12	11.89 ± 0.13	12.22 ± 0.17	4.99	0.01*
Eruption of lower incisors	10.11 ± 0.15	10.24 ± 0.19	10.14 ± 0.14	10.31 ± 0.15	0.33	0.81
Eyes openning	14.64 ± 0.17	14.57 ± 0.11	14.57 ± 0.34	14.55 ± 0.19	0.03	0.99
**Neurobehavioral development**						
Negative Geotaxis	3.61 ± 0.19	3.70 ± 0.17	3.88 ± 0.18	3.81 ± 0.17	0.46	0.71
Cliff Aversion	3.20 ± 0.05	3.24 ± 0.05	3.50 ± 0.16	3.33 ± 0.15	2.04	0.19
Open Field Traversal	9.50 ± 0.41	9.17 ± 0.15	9.84 ± 0.22	9.58 ± 0.28	1.44	0.26

All data were expressed as mean ± SEM.

* P<0.05.

The effects of maternal fenvalerate exposure during pregnancy on the neurobehavioral development of neonatal mice were also investigated during location period. As shown in Table 1, there was no significant difference on negative geotaxis, cliff aversion and open field traversal in offspring dams among different groups.

### Effects of maternal fenvalerate exposure during pregnancy on anxiety-related behaviors in mouse offspring

**Fig 3:**
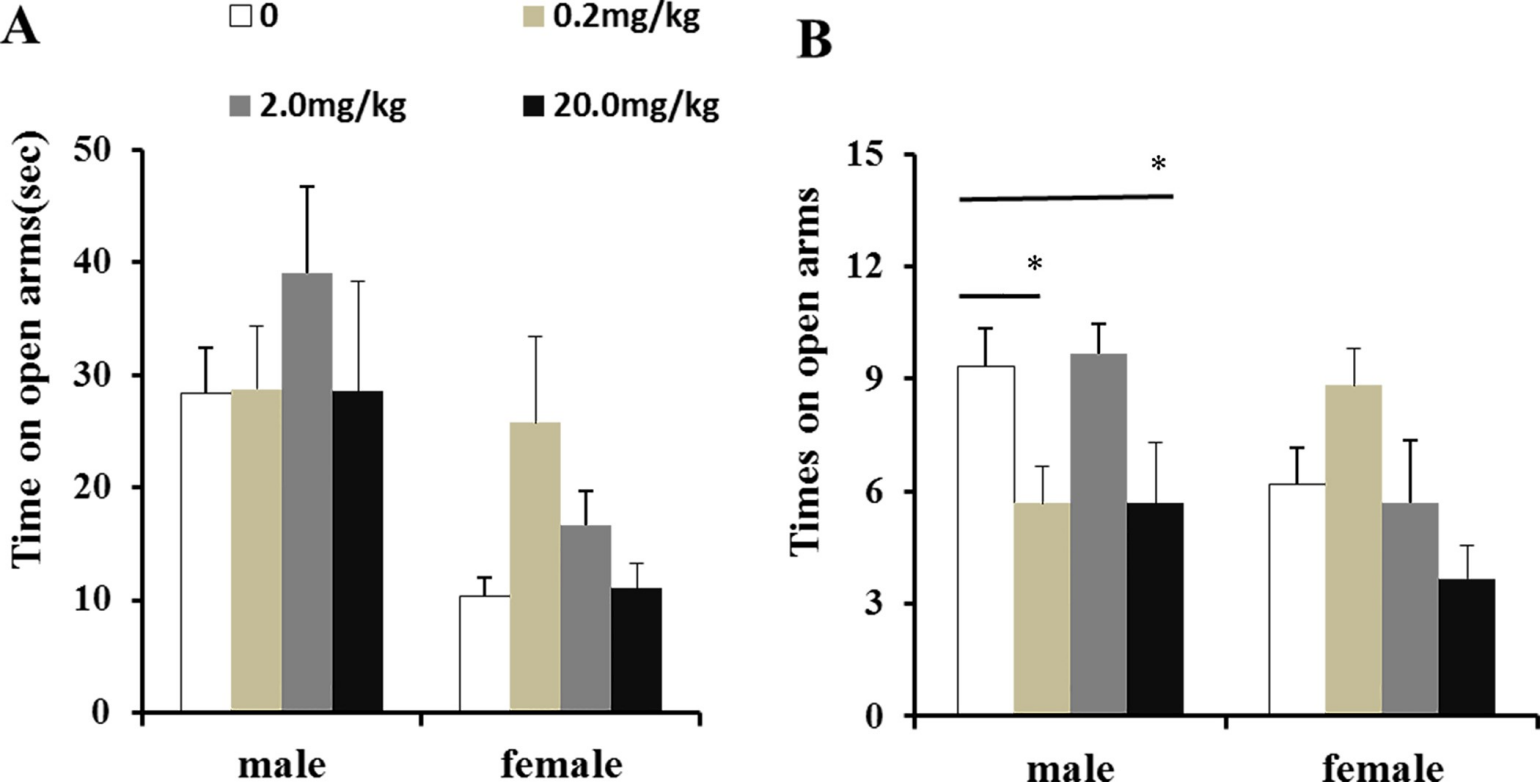
Effects of maternal fenvalerate exposure on anxiety-related behaviors of offspring in elevated plus maze. Pregnant mice were administered daily with fenvalerate (0, 0.2, 2.0, and 20 mg/kg) by gavage from GD0 to GD18. Elevated plus maze was performed on PND40. (A) Time on open arms. (B) Times on open arms. All data were presented as mean± SEM. *P<0.*05* compared with the controls.

The effects of pregnant exposure to fenvalerate on anxiety-related behaviors were examined by elevated plus-maze and open-field. In the elevated plus-maze, no significant difference in time on open arms was observed among different groups (Fig 3A). However, a significant difference in times on open arms was observed between fenvalerate-exposed offsping and controls. Further analysis showed that it was only in male offspring, not in female offspring, times on open arms were significantly decreased by exposed to 0.2 mg/kg and 20 mg/kg fenvalerate (Fig 3B, *P<0*.*05*). In the open-field test, as shown in Table 2, the number of feces and rearing/leaning in male dams were significantly increased, though no statistical difference on square across, total distance moved, in center duration and grooming among different groups. As expected, no significant difference was observed in female offspring among groups (Table 2).

**Table 2:** Effects of maternal fenvalerate exposure during pregnancy on anxiety-related behaviors in mouse offspring.

	0	0.2mg/kg	2.0mg/kg	20.0mg/kg	*F*	*P*
**Male**						
Squares across	66.4 ± 5.8	75.3 ± 4.6	83.0 ± 5.9	73.6 ± 4.7	1.67	0.21
Total distance moved(m)	11.7 ± 0.9	13.9 ± 0.8	14.4 ± 1.2	13.6 ± 0.9	1.55	0.23
In center duration(s)	10.9 ± 2.6	11.3 ± 2.3	12.1 ± 1.4	8.7 ± 1.4	0.56	0.65
Number of feces	1.3 ± 0.4	1.6 ± 0.5	2.2 ± 0.2	3.3 ± 0.4	5.67	0.01*
Grooming	1.0 ± 0.3	2.0 ± 0.5	1.4 ± 0.2	1.7 ± 0.5	1.09	0.38
Rearing/leaning	27.8 ± 1.2	36.6 ± 3.3	39.8 ± 2.7	39.8 ± 4.5	3.32	0.04*
**Female**						
Squares across	68.5 ± 2.3	77.7 ± 7.2	86.9 ± 6.6	79.3 ± 10.2	2.02	0.14
Total distance moved(m)	12.8 ± 0.5	12.2 ± 1.1	15.5 ± 1.0	14.8 ± 1.7	1.00	0.41
In center duration(s)	10.1 ± 2.5	9.9 ± 1.4	8.5 ± 1.1	9.6 ± 0.8	0.12	0.95
Number of feces	2.3 ± 0.7	2.3 ± 0.7	2.7 ± 0.9	3.8 ± 0.9	0.78	0.52
Grooming	0.9 ± 0.2	1.0 ± 0.3	1.6 ± 0.2	1.1 ± 0.3	1.25	0.32
Rearing/leaning	37.5 ± 2.1	36.8 ± 3.6	42.5 ± 3.0	44.8 ± 3.0	2.02	0.14

The Open field was carried on PND35. The data was present as mean± SEM.

* P<0.05.

### Effects of maternal fenvalerate exposure during pregnancy on spatial learning and memory in mouse offspring

**Fig 4:**
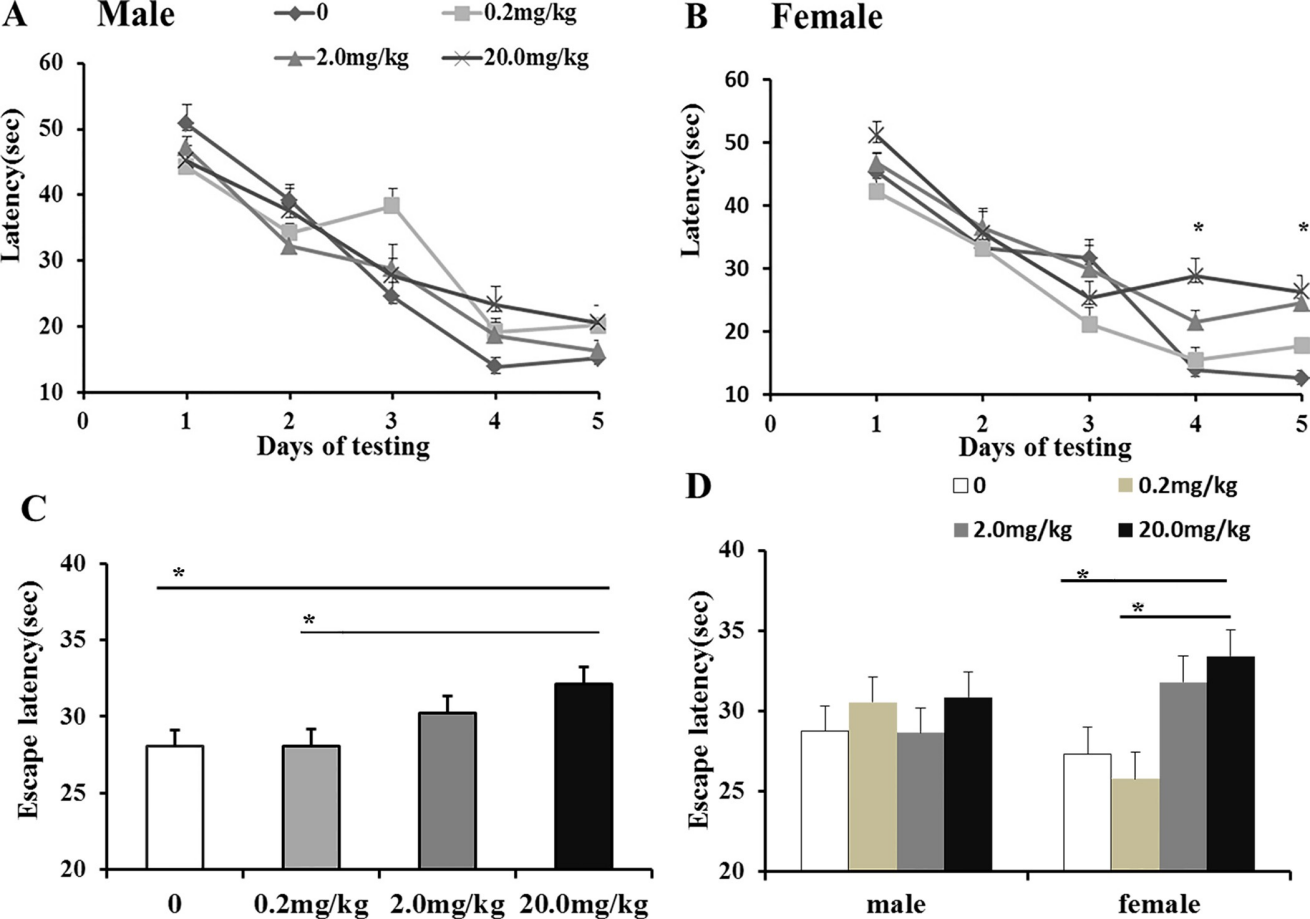
Effects of maternal fenvalerate exposure on escape latency of spatial learning performance of mouse offspring. Pregnant mice were administered with fenvalerate (0, 0.2, 2.0, and 20 mg/kg) by gavage daily from GD0 to GD18. Place navigation trial of Morris water maze was performed from PND45 to PND49. (A) Escape latency of male offspring with the day of learning (two- way ANOVA). (B) Escape latency of female offspring with the day of learning (two- way ANOVA). (C) Escape latency of all dames (repeated measures ANOVA). (D) Escape latency of males and females (repeated measures ANOVA). All data was presented as mean± SEM. *P<0.*05* compared with the controls.

**Fig 5:**
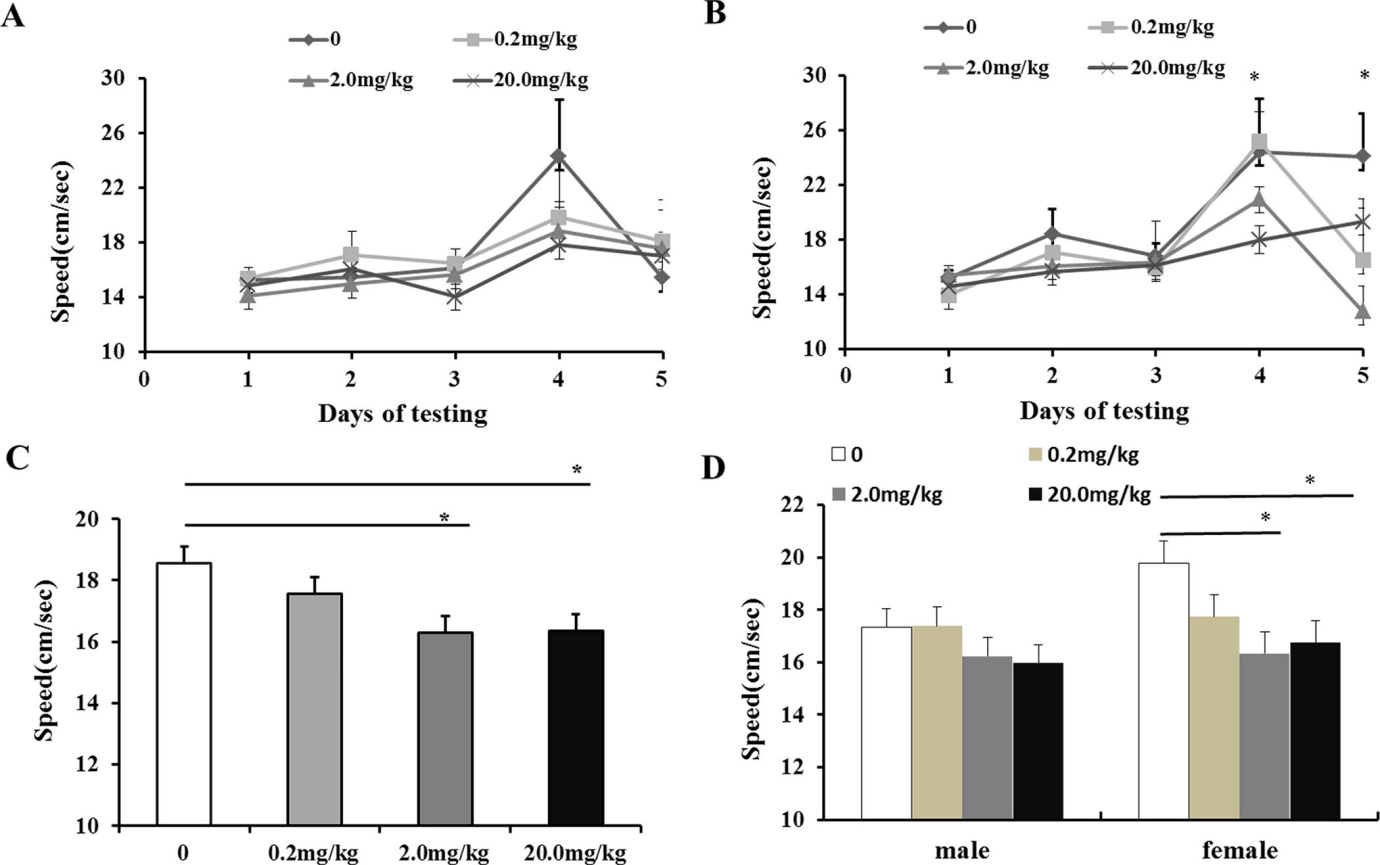
Effects of maternal fenvalerate exposure on swim speed of spatial learning performance of mouse offspring. Pregnant mice were administered with fenvalerate (0, 0.2, 2.0, and 20 mg/kg) by gavage daily from GD0 to GD18. Place navigation trial of Morris water maze was performed from PND45 to PND49. (A) Swim speed of male offspring with the day of learning. (B) Swim speed of female offspring with the day of learning. (C) Swim speed of all offspring. (D) Swim speed of male and female offspring. All data were presented as mean± SEM. *P<0.*05* compared with the controls. (A and B, two- way ANOVA; C and D, repeated measures ANOVA).

The effects of maternal fenvalerate exposure during pregnancy on learning and memory performances were detected by Morris Water Maze. As showed in Fig 4A and 4B, compared with control group, a significant difference in escaping latency of fenvalerate-treated female mice was observed on day 4 and 5, while no significant difference was observed in male mice. Repeated measures indicated that a significant difference in escaping latency of female mice was observed and that the escaping latency was longer in fenvalerate-treated group than control group (Fig 4C and 4D, *P*<0.05). In consistent with escaping latency, swim speed of fenvalerate-treated female mice was slower than controls (Fig 5A, 5B, 5C and 5D, *P*<0.05).

**Fig 6:**
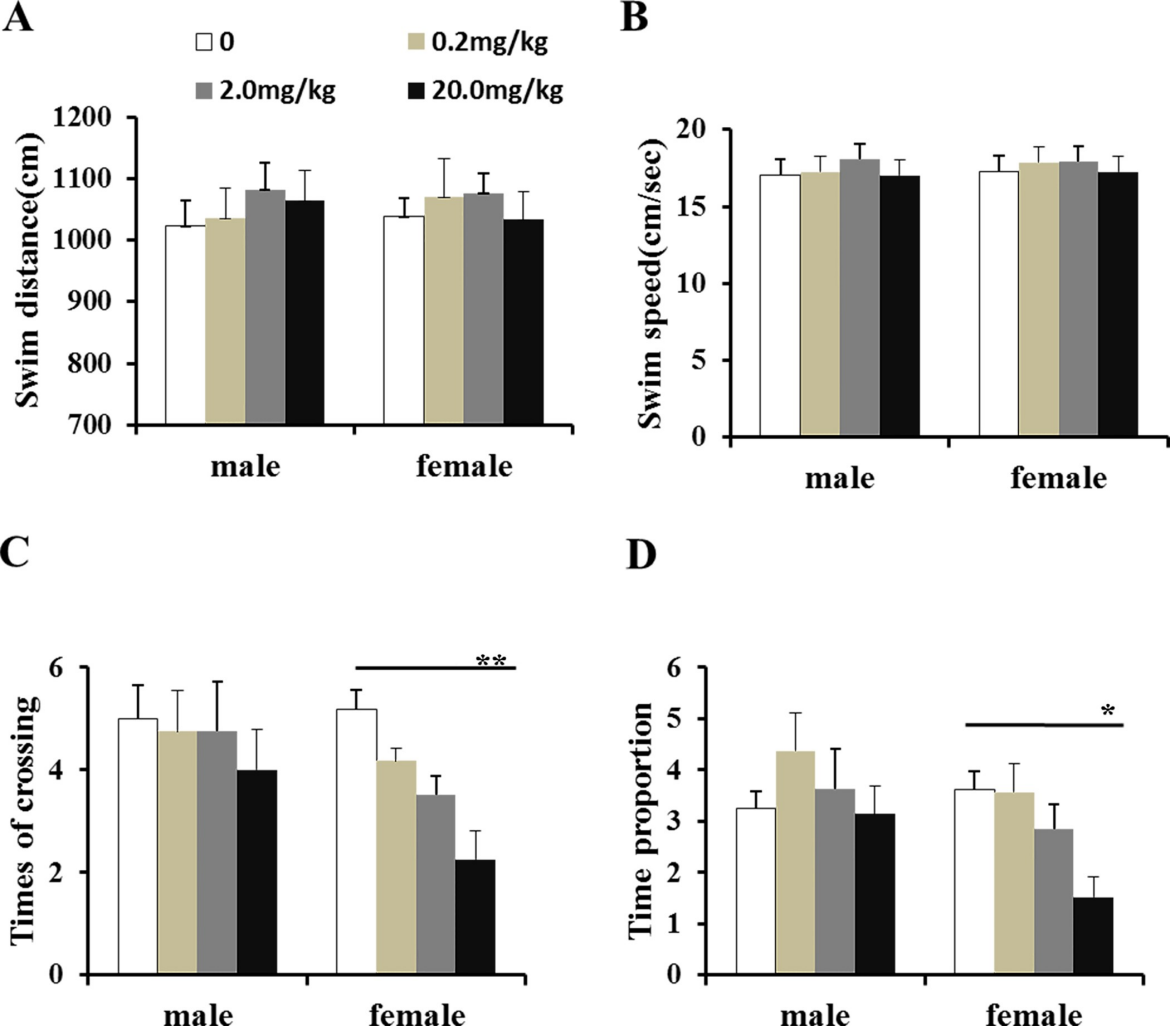
Effects of maternal fenvalerate exposure during pregnancy on spatial memory performance of mouse offpring. Pregnant mice were administered daily with fenvalerate (0, 0.2, 2.0, and 20 mg/kg) by gavage from GD0 to GD18. Spatial probe trial of Morris water maze was performed on PND50. (A) Swim distance of objective quadrant. (B) Swim speed of objective quadrant. (C) Times of crossing of objective quadrant. (D) Time proportion of objective quadrant. All data were presented as mean± SEM. *P<0.*05*, **P< 0.*01* compared with the controls.

Spatial memory was examined on the sixth day. No significant difference was observed on swimming distance and swimming speed among different groups (Fig 6A and 6B). Interestingly, significant differences in frequency of crossing and time proportion of objective quadrant were was only observed in female mouse (Fig 6C and 6D). Further analyses showed that both times of crossing and time proportion of objective quadrant were markedly decreased in female mice treated with fenvalerate at dose of 20 mg/kg (Fig 6C and 6D).

## Discussion

Previous study from our laboratory showed that fenvalerate-exposure during puberty impaired spatial cognitive and behavioral development [8]. The present study further found that maternal exposure to fenvalerate during pregnancy delayed growth and neurobehavioral development of mouse offspring in a gender-dependent manner. First, maternal exposure to fenvalerate markedly delayed growth of mouse offspring during lactation period. Second, we assessed neurobehavioral development from PND35 to PND50. We found that anxiety-related behaviors were increased in fenvalerate-treated male offspring. By contrast, spatial learning and memory was markedly impaired in female offspring, not in male offspring. As far as we know, this was the first study to evaluate the effects of maternal exposure to fenvalerate during pregnancy on growth and neurobehavioral development in mice offspring.

Over several decades, growing evidence demonstrated that some chronic diseases, comprising neurodevelopmental disorders, might be traced back to environmental exposures within the uterus [19]. Experts have named this emerging paradigm as “developmental origins of health and disease (DOHaD)” [20–21]. An increasing number of epidemiological studies and animal models have similarly reported an adverse link between prenatal exposure to pesticides and neurodevelopment in children. For example, a study in California utilizing the CHAMACOS cohort found that prenatal exposure to pesticides may delay cognitive development in children [22]. Other cohort studies found a potential adverse link between maternal residential proximity to agricultural use of neurotoxic pesticides and neurodevelopment of children [23–24]. However, an epidemiological report found that postnatal fenvalerate exposure was not related to learning disability and attention deficit/hyperactivity disorder in U.S. children [25]. The present results showed that maternal exposure to fenvalerate during pregnancy not only prolonged physiology development, but also increased anxiety and impaired spatial cognition. Future research should assess the safety of current levels of fenvalerate, especially in intrauterine exposure.

Numerous data have demonstrated that there are sex differences on brain and behaviors [26–27]. A recent study showed that there was more severe cognitive impairment in girls than in boys who exposed to insecticide during prenatal period [22]. An early report from our laboratory showed that the impairment of spatial learning and memory was more serious in female mice than male mice exposed to fenvalerate during puberty [8]. Being consistent with the previous studies, the present study showed that spatial learning and memory were significantly impaired in female offspring, but not in male offspring. Interestingly, we found that maternal fenvalerate exposure significantly increased anxiety-related behaviors in male dams, not in females. By contrast, pubertal fenvalerate exposure interferes with anxiety-like behaviors only in female mice [8]. These data showed that there was opposite gender-dependent manners in anxiety-related behaviors which induced by fenvalerate-exposure during pregnancy or puberty, critical windows of development. Further study should be focused on underlying mechanism of this inconsistence of gender-dependent manners.

The mechanism by which maternal fenvalerate exposure impairs neurodevelopment remains obscure. Recently, endocrine disruption has emerged as a possible mechanism [28]. Fenvalerate acts as an EDC [29]. Our recent study found that maternal exposure to fenvalerate during pregnancy decreased the expression of placental TRα1 and TRβ1 [12]. Earlier results from our laboratory showed that fenvalerate exposure during puberty disrupted T and E2 synthesis in cerebral cortex [11]. In addition, pubertal fenvalerate exposure disrupted the expression of AR in cerebral cortex [11]. It is well known that hormone (such as T, E2 and TR) plays an important role in cognitive function and behavioral development. These data suggest that fenvalerate-induced impairment on cognition and behavioral development might be associated with endocrine disruption. However, other mechanisms cannot be ruled out. Indeed, fenvalerate is also a kind of developmental toxicant. Our recent animal research also found that maternal exposure to fenvalerate during pregnancy resulted in fetal intrauterine growth restriction (IUGR) [12]. An epidemiology study reported prenatal exposure to fenvalerate was linked to birth weight in Rural Northern China [30]. Moreover, fenvalerate also induced developmental neurotoxicity [31]. Taken together, additional study is essential to explore the exact mechanism by which fenvalerate-exposure impairs growth and neurobehavioral development.

There were three main advantages in the present study. First, we chose pregnant period as exposure time, considering the emerging paradigm named as “developmental origins of health and disease (DOHaD)”. Second, we assess its long effects from location to early stage of life. Third, the present study found a gender-dependent manner in which maternal exposure to fenvalerate impaired neurobehavioral development. However, there were some weaknesses in the present study. We did not assess the effects of maternal exposure to fenvalerate during pregnancy on hormone levels in the brain and blood of offspring. In addition, our study did not investigate the mechanism by which maternal fenvalerate exposure delayed growth and neurobehavioral development of mouse offspring.

In summary, this was the first study to evaluate maternal exposure to fenvalerate during pregnancy on growth and neurobehavioral development in mice offspring. The results from our study showed that maternal exposure to fenvalerate during pregnancy significantly delayed growth of neonatal offspring during lactation period. In addition, we found that anxiety-like behaviors were increased in male offspring. Moreover, spatial learning and memory were significantly impaired in female offspring. Taken together, maternal exposure to fenvalerate during pregnancy delayed growth and neurobehavioral development of mouse offspring in a gender-dependent manner. Additional study is essential to explore the underlying mechanism of maternal exposure to fenvalerate during pregnancy on growth and neurobehavioral development.

## Supporting information

S1 Table Experimental data. (XLS)
